# Dual targeting of cancer-related human matrix metalloproteinases and carbonic anhydrases by chiral *N*-(biarylsulfonyl)-phosphonic acids

**DOI:** 10.1080/14756366.2017.1378192

**Published:** 2017-09-26

**Authors:** Grazia Luisi, Guido Angelini, Carla Gasbarri, Antonio Laghezza, Mariangela Agamennone, Fulvio Loiodice, Claudiu T. Supuran, Cristina Campestre, Paolo Tortorella

**Affiliations:** a Department of Pharmacy, “G. d’Annunzio” University of Chieti-Pescara, Chieti, Italy;; b Department of Pharmacy and Pharmaceutical Sciences, “A. Moro” University of Bari, Bari, Italy;; c Neurofarba Department, Section of Pharmaceutical and Nutriceutical Sciences, University of Florence, Sesto Fiorentino (Florence), Italy

**Keywords:** Carbonic anhydrases, matrix metalloproteinases, phosphonic acids, sulfonamides, zinc-binding group

## Abstract

A series of nanomolar phosphonate matrix metalloproteinase (MPP) inhibitors was tested for inhibitory activity against a panel of selected human carbonic anhydrase (CA, EC 4.2.1.1) isozymes, covering the cancer-associated CA IX and XII. None of the reported sulfonyl and sulfonylamino-derivatives sensitively affected the catalytic activity of the cytosolic isoforms CA I and II, which are considered off-target isoforms in view of their physiological role. The most active inhibitors were in the series of chiral *N*-(sulfonyl)phosphovaline derivatives, which showed good to excellent inhibitory activity over target CAs, with compound **15** presenting the best isoform-selectivity toward CA IX. We suggest here that the phosphonates have the potential as dual inhibitors of MMPs and CAs, both involved in tumor formation, invasion and metastasis.

## Introduction

Carbonic anhydrases (CAs, carbonate hydro-lyases, EC 4.2.1.1) are a large family of metalloenzymes that primarily act as efficient catalyzers of the reversible hydration of carbon dioxide to bicarbonate ions and protons^1–3^. This biochemical reaction has been recognized a crucial role in regulating CO_2_/electrolite interchange at the respiratory and photosynthetic surfaces in eukaryotic as well prokaryotic organisms^4^, with a corollary of diverse physiological processes depending on this activity, including pH homeostasis, ion transport, bone dynamics, and energetic metabolism^4^.

It is widely accepted that a deregulation in CA function and expression is involved in the pathogenesis of important human diseases, among all glaucoma, epilepsy, osteoporosis, and cancer^5–7^. A growing attention is focused on both CA IX and CA XII, since these trans-membrane isoenzymes have been reported to be overexpressed in many tumors, and associated with cancer progression and metastasis. In order to survive to the hypoxic stress, tumor cells execute an aberrant metabolic adjustment known as the Warburg effect^8^, which leads to acidification of the extracellular *milieu*; this harsh micro-environment creates selective survival conditions for solid tumors over normal tissues, by facilitating cancer invasiveness. The need to maintain the intracellular pH near physiological levels requires the activation of complex molecular mechanisms, such as the up-regulation of CA IX and CA XII. Therefore, these isoforms have become attractive targets for anti-cancer therapeutic purposes^9^^,^^10^. It is worthy to note that, unlike CA XII, CA IX is minimally expressed in normal tissues. Furthermore, compared with isoform XII, CA IX is more prevalent in solid and metastatic tumors, the ones that more frequently develop resistance to radiation and/or chemotherapy, probably because this isoform is able to retain its catalytic activity even in the acidic conditions of the tumor habitat.

Thus, the inhibition of this latter enzyme represents one of the most opportune strategy to sensitize highly aggressive tumors to the effects of antineoplastic drugs. From a general point of view, however, the single target approach in drug discovery often results in a poorer therapeutic impact if compared to the synergic effects obtainable with the modulation of multiple systems; thus, we were intrigued in the development of dual-acting ligands for cancer-related enzymes that, although belonging to different families, and despite the different mechanism of action, share significant commonalities in terms of molecular recognition. Over the years, we focused our attention on matrix metalloproteinases (MMPs), a family of zinc-dependent isoenzymes involved in bone homeostasis, extracellular matrix remodeling, and inflammation^11^. Dysregulated MMPs, particularly MMP-2, have long been recognized to have a crucial role in tumor genesis and aggressiveness^12^, giving rise to the challenging approach of inhibiting chosen MMPs for cancer treatment^13^^,^^14^.

Recently, we capitalized on the observation that both MMP and α-class CA proteins depend upon the presence of a zinc ion in the catalytic domain to process substrates, as starting point to establish if the two enzymatic families could be inhibited by similar compounds. We reported a variety of potent and selective MMP inhibitors (MMPIs) based on the presence of a zinc-binding group (ZBG) coupled to a variable backbone able to fit into the S1’ specificity pocket of the individual MMP active site^15–17^. Due to its bidentate nature, the hydroxamic acid is one of the most strong chelating group for catalytic metal ions, and indeed the hydroxamate-based MMP inhibitors have been found the most active *in vitro*; however all attempts to obtain clinically successful drugs from inhibitors featuring this moiety have failed owing to common drawbacks, such as low MMP subtype selectivity, activity on a broad range of metal ions, including Fe(III), *in vivo* instability, dose limiting side effects and low oral availability^18^^,^^19^. To address the main limitations of hydroxamates, alternative monodentate ZBGs such as carboxylate, thiolate, and phosphonate, that coordinate rather than chelate the zinc ion, may be used in metalloenzyme inhibitor design to obtain a gain in inhibitor selectivity; owing to the formation of less stable metal coordination complexes, in fact, a major contribute to the binding between inhibitor and protein is played by the distinctive substituents on the molecule scaffold^20^.

This approach has been successfully exploited in our laboratories, leading to a series of (*R*)-α-biarylsulfonylamino-2-methyl-propyl phosphonates as MMPIs characterized by high potency and enantioselectivity towards a panel of target isoforms;^16^^,^^17^ they represent by far the most powerful inhibitors containing the phosphonate moiety as ZBG, exhibiting IC_50_ values in the nanomolar or even sub-nanomolar range. Our MMP-8 selective inhibitor (*R*)-1-[3′-methyl-biphenyl-4-sulfonylamino]-2-methylpropyl phosphonic acid has been shown to play an *in vivo* protective effect in experimental models of multiple sclerosis, systemic inflammatory response syndrome, and ventilator-induced lung injury, thus corroborating the link between MMP-8 up-regulation and systemic inflammatory diseases^21–23^.

For the purpose to obtain further insights on this topic, we then investigated a small set of analogs of the reported α-arylsulfonylamino-methyl phosphonates, characterized by the replacement of the sulfonylamino with a sulfonyl group in alpha to the ZBG. Although most of the new α-arylsulfonyl-methyl phosphonates were found good to excellent inhibitors of selected MMPs, they resulted less active in comparison with their sulfonylamino counterparts; however, these phosponates are notably more selective for MMP-2 over the anti-target isoforms MMP-3 and MMP-9^24^.

We were then intrigued in evaluating a panel of compounds from these two chemical entities as CA inhibitors, with the aim to disclose a possible dual activity against MMPs and CAs. To the best of our knowledge, no data on dual MMP/CA inhibitors containing either sulfonylaminomethyl- or sulfonylmethyl-groups directly bound to a single phosphonate ZBG have been reported to date; in this context only related studies, disclosing sulfonylaminomethyl- or arylaminomethyl- bisphosphonates,^25^^,^^26^ and sulfonylated-aminoacyl-hydroxamates^27^ as dual inhibitors, have been published by our team.

The α-arylsulfonylamino-methyl phosphonates under scrutiny (compounds **12–15**) contain a secondary sulfonamide moiety (-SO_2_NH-), a distinctive feature of classical CAIs which bind as monodentate anions to the zinc ion in the active site, displacing the Zn^2+^-bound solvent molecule. Although multiple alternative inhibition mechanisms have been evidenced, as thoroughly detailed in our expertise,^28^^,^^29^ the sulfonamide-based CA inhibitors remain between the most successful drug candidates. The α-arylsulfonyl-methyl phosphonates we tested in the present study (compounds **1, 4–11**) exhibit a sulfonyl (-SO_2_-) unit, and are therefore devoid of the possible second coordinating group. For comparison, the α-arylsulfonyl-methyl carboxylate and hydroxamate analogs (compounds **2** and **3**, respectively) have been also investigated.

It should be mentioned that compounds **12–15** can be considered under all respects as *N*-(biphenylsulfonyl)-phosphovaline derivatives, in that they contain the phosphonic counterpart of aminoacid valine. By testing chiral **12** and **15**, which incorporate a residue of (*R*)-phosphovaline, we intended to evaluate the impact of isopropyl side-chain orientation on CA inhibitory activity and selectivity.

## Experimental

The synthetic protocol for compounds **1–15** has been previously reported^16^^,^^17^^,^^24^.

### CA inhibition

An applied photophysics stopped-flow instrument has been used for assaying the CA catalyzed CO_2_ hydration activity. Phenol red (at a concentration of 0.2 mM) has been used as indicator, working at the absorbance maximum of 557 nm, with 20 mM Hepes, pH 7.5, as buffer, and 20 mM NaClO_4_ (for maintaining constant the ionic strength), following the initial rates of the CA-catalyzed CO_2_ hydration reaction for a period of 10–100 s. The CO_2_ concentrations ranged from 1.7 to 17 mM for the determination of the kinetic parameters and inhibition constants. For each inhibitor at least six traces of the initial 5–10% of the reaction have been used for determining the initial velocity. The uncatalyzed rates were determined in the same manner and subtracted from the total observed rates. Stock solutions of inhibitor (10–50 mM) were prepared in distilled-deionized water and dilutions up to 0.1 nM were done thereafter with the assay buffer. Inhibitor and enzyme solutions were preincubated together for 15 min at room temperature prior to assay, in order to allow for the formation of the E–I complex or for the eventual active site mediated hydrolysis of the inhibitor. The IC_50_ values were obtained by non-linear least-squares methods using PRISM 3 and represent the molarity of inhibitor at which the enzyme activity was halved (mean from at least three different determinations). Human CA I, II, IX, XII, and XIV were recombinant proteins obtained in-house as described earlier^30–37^.

## Results and discussion

The inhibitory properties of the previously identified MMP inhibitors **1–15** have been investigated on five CA members, and more specifically against the cytosolic human CA I and II, which are widespread throughout the human body and represent drug targets for clinically used diuretics, antiglaucoma drugs, and anticonvulsants, as well as on the trans-membrane human isoforms CA IX and XII, that are validated antitumor targets, and XIV, which has been described as a possible objective for the treatment of epilepsy, some retinopathies, and skin tumors^31^. The standard, clinically used, CA inhibitor acetazolamide (AAZ) has been used as reference compound.

The initial investigation of compounds **1–15** on CA I and II meets the purpose to assay the novel inhibitory activity of this set of phosphonate-based MMPIs against cytoplasmic CAs, and to evaluate the most active compounds against trans-membrane CAs, in order to obtain a more complete inhibitory profile. This is congruent, in view of the high degree of sequence and structural homology at the sites relevant to catalysis in all mammalian CA isoforms.

The *in vitro* inhibition effects of derivatives **1–15** on CA isoforms I and II are outlined in Table 1. All compounds resulted inactive on the ubiquitous CA I, with only the hydroxamic derivative **3** showing a very weak activity against this isozyme (IC_50_ = 8.3 μM). A better inhibitory profile was observed on CA II, but only for compounds **3**, **12**, and **15**, which resulted micromolar inhibitors of the enzyme, although with an almost 100-fold decrease in activity compared to the standard AAZ.

**Table 1.** Inhibition data for compounds **1–15** against CAs.


						IC_50_/μm^a^	
Compound	R	R′	R″	X	ZBG	CA I	CA II
**AAZ**						0.299	0.023
1	C_6_H_5_	H	H	SO_2_	PO_3_H_2_	>10	>10
2	C_6_H_5_	H	H	SO_2_	COOH	>10	>10
3	C_6_H_5_	H	H	SO_2_	CONHOH	8.299	3.660
4	4-CH_3_O-C_6_H_4_	H	H	SO_2_	PO_3_H_2_	>10	>10
5	4-CF_3_-C_6_H_4_	H	H	SO_2_	PO_3_H_2_	>10	>10
6	3-Cl-C_6_H_4_	H	H	SO_2_	PO_3_H_2_	>10	>10
7	2-thienyl	H	H	SO_2_	PO_3_H_2_	>10	>10
8	4-CH_3_O-C_6_H_4_O	H	H	SO_2_	PO_3_H_2_	>10	>10
9	C_6_H_5_	H	(CH_3_)_2_CHCH_2_	SO_2_	PO_3_H_2_	>10	>10
10	4-CH_3_O-C_6_H_4_	H	C_6_H_5_CH_2_	SO_2_	PO_3_H_2_	>10	>10
11	C_6_H_5_	CH_3_	CH_3_	SO_2_	PO_3_H_2_	>10	>10
(*R*)-12	4-CH_3_-C_6_H_4_	H	(CH_3_)_2_CH	SO_2_NH	PO_3_H_2_	>10	4.140
(*R*)-13	3-CH_3_-C_6_H_4_	H	(CH_3_)_2_CH	SO_2_NH	PO_3_H_2_	>10	>10
14	4-Br-	H	(CH_3_)_2_CH	SO_2_NH	PO_3_H_2_	>10	>10
(*R*)-15	4-CH_3_O-C_6_H_4_	H	(CH_3_)_2_CH	SO_2_NH	PO_3_H_2_	>10	3.777

a Errors in the range of 5–10% of the reported value (from three different assays).

The most active compounds **3**, **12**, **15** were selected for subsequent evaluation on CA IX, XII and XIV. Sulfonyl derivative **10**, sharing the SAR-controlling 4′-CH_3_O- substituent on the biphenyl group with the sulphonylammino-phosphonate **15**,^17^^,^^24^ was investigated as well on the cited isoforms, although essentially inactive on CA I and II. Table 2 collects the observed inhibitory activities of such compounds on the complete group of CAs, together with the existing data on MMP-2 and MMP-8^16^^,^^17^^,^^24^.

**Table 2.** Inhibition data for compounds **3**, **10**, **12**, and **15** against CAs and MMPs.


					IC50/μm^a^						
Compound	R	R″	A	ZBG	CA I	CA II	CA IX	CA XII	CA XIV	MMP-2	MMP-8
**AAZ**					0.299	0.023	0.903	0.088	0.224	ND	ND
3	H	H	–	CONHOH	8.299	3.660	0.527	8.862	8.525	0.0060	0.024
10	4-CH_3_O	C_6_H_5_CH_2_	–	PO**_**3**_**H**_**2**_**	>10	21.102	0.837	8.483	7.472	8.2	ND
(R)-12	4-CH_3_	(CH_3_)_2_CH	NH	PO**_**3**_**H**_**2**_**	>10	4.140^b^	0.093	0.475	0.369	0.0023	0.0004
(R)-15	4-CH_3_O	(CH_3_)_2_CH	NH	PO**_**3**_**H**_**2**_**	>10	3.777	0.084	6.220	1.521	0.0015	0.0014

a Errors in the range of 5–10% of the reported value (from three different assays).

b CA II from Sigma. pedices have been corrected.

The potent MMP inhibitor of the hydroxamate-type **3** displayed only a one-digit micromolar inhibition on cytosolic isoforms CA I and II and membrane-bounds CA XII and XIV, but achieved its maximal potency on CA IX, with a sub-micromolar IC_50_ that resulted almost half the value of the reference compound AAZ on the same isoform (0.5 vs. 0.9 μM, respectively).

Compound **10** resulted less active than the hydroxamic derivative **3** against MMP-2 and CAs; however, this sulfonyl derivative, with an IC_50_ against CA IX in the high nanomolar range, that is almost 10 or even 20 (see CA II) times lower than the corresponding values for the remaining CAs, manifested a certain selectivity towards this isoform.

Sulfonylamino-derivatives **12** and **15**, while exhibiting an activity comparable to that of hydroxamate **3** on the cytosolic isozymes, presented a quite interesting inhibition profile against CA IX, with corresponding IC_50_ values in the low nanomolar range. Furthermore **12** was able to inhibit, albeit with a 4-fold decrease in potency in comparison to CA IX, the other two isoforms CA XII (IC_50_ = 0.47 μM) and CA XIV (IC_50_ = 0.37 μM), thus resulting the most complete CA/MMP inhibitor. Compared to **12**, its (*R*)-*N*-(4′-methoxy) analog **15**, differing only for the CH_3_O-substituent in the 4′ position of the biphenyl unit instead of the CH_3_ group, manifested weaker potencies on CA XII and XIV, but shared the low nanomolar inhibitory activity on CA IX, thus showing selectivity towards this latter isoform.

With respect to the inhibitory profiles of tested compounds **1–15**, the following observations can be pointed out: with the exception of the α-biphenylsulfonyl-methyl hydroxamate **3**, which inhibited CA I and II in the low micromolar range, the α-biphenylsulfonylmethyl carboxylic acid **2** and all the other substituted α-biarylsulfonylmethyl phosphonates (compounds **1**, **4–11**) were completely devoid of activity towards these off-target isoforms, regardless of the nature of the substituents. In spite of this, racemic 1–(4′-methoxybiphenyl-4-sulfonyl)-2-phenylethylphosphonic acid (**10**) was considered for inhibition of the three membrane-bound CAs, resulting in the more selective CA IX phosponate inhibitor of the series.

These experimental results could be tentatively analyzed in light of the assumption that none of the two oxygen atoms of the sulfonyl moiety is able to establish hydrogen bond interactions so productive as to reinforce the Zn^2+^-coordination network from the dissociated phosphonic (compounds **1**, **4–11**) or carboxylic acid group (compound **2**) inside the CA I and II active sites. As far as the α-biphenylsulfonyl-methyl hydroxamate **3** is concerned, we postulated that the stronger binding properties of the bidentate -CONHOH chelator may account for the appearance of the moderate to good inhibition observed towards the full panel of selected CAs. The expected lack of selectivity and the lipophylic character of hydroxamate **3**, on the other hand, were the main limitations we tried to deal with by introducing alternative, but clearly weaker, ZBGs in the sulfonyl series.

The nanomolar MMPIs (*R*)-*N*-(4′-methylbiphenylsulfonyl)-phosphovaline (**12**) and (*R*)-*N*-(4′-methoxybiphenylsulfonyl)-phosphovaline (**15**) manifested excellent activities also on target-CAs, and particularly on CA IX. They both feature a secondary –SO_2_NHR fragment positioned next to the ZBG, which possess good metal-coordinating properties; so we expect that both the sulfonamide and the phosphonate contribute to ion binding in a bidentate mode, somewhat in analogy to the model we proposed for the sulfonylaminoacyl-hydroxamate dual-inhibitors of the same enzyme families^27^. With an inhibitory profile ranging from the sub-nanomolar IC_50_ value against MMP-8 to the two/three digit nanomolar activities towards target CAs, compound **12** resulted the most powerful inhibitor in this set, thus presenting a potential in view of its cross-reactivity on cancer-related MMP/CA members.

## Conclusions

Dysregulated expression of membrane-bound CA IX and XII results in decrease of extracellular pH, an event which is strongly associated with cancer cell survival and malignant progression. Thus specifically targeting these tumor-associated isoforms over the cytosolic CA I and II, which have a physiological relevance, is considered to be a promising strategy in cancer therapy. In recent years, multitarget approaches directed toward inhibition of CAs and enzymes of different families have received increasing attention. The MMPs are a family of metallo-endopeptidases that play a central role in many important physiological and physiopathological events, including tumor invasion and metastasis. Taking advantage of the fact that MMPs and CAs are both zinc-dependent enzymes, the approach of developing molecules with the ability to inhibit selected members of both families is particularly challenging.

In this article, we describe the inhibitory properties of a series of potent phosphonate-based MMP inhibitors against the cytosolic off-target isoforms CA I and II and the three trans-membrane (two of which are cancer-related) isoforms CA IX, XII and XIV. The most active inhibitors are found in the series of chiral *N*-(sulfonyl)phosphovaline-derivatives, which manifest good to excellent inhibitory activity over target CAs, with compound **15** showing the best isoform-selectivity toward CA IX. We identified compound **12** as our best inhibitor in view of its high and broad efficacy on the selected metalloenzymes: the ability of this small molecule to interact with different targets involved in cancerogenesis may be beneficial to address drawbacks of the classic anti-cancer protocol. Again, activity data confirm the peculiar role of the SO_2_NH functionality in stereoelectronic recognition and binding to the metal-core of the active site.

Despite the structural and catalytic differences of these two enzyme superfamilies, it is evident that MMP and CA active sites may be targeted by common, privileged structures. Closer investigations with the aim of understanding the overlapping chemical space of these target proteins will be necessary to further explore the dual-inhibition approach.
